# Supplementary figures and images for: Impaired emotion recognition is linked to alexithymia in heroin addicts
Source: PeerJ. 2016 Apr 5;4:e1864. doi: 10.7717/peerj.1864 (PMC4824882; doi:10.7717/peerj.1864)

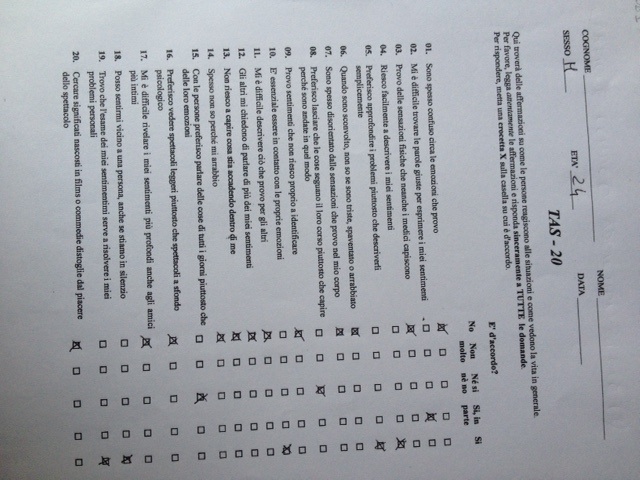

Supplement: Data S1 [file peerj-04-1864-s001.zip › gruppo clinico/IMG_3247.jpg]

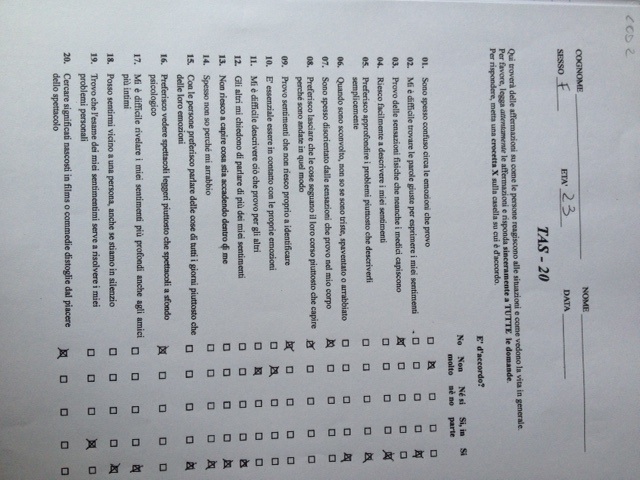

Supplement: Data S1 [file peerj-04-1864-s001.zip › gruppo clinico/IMG_3248.jpg]

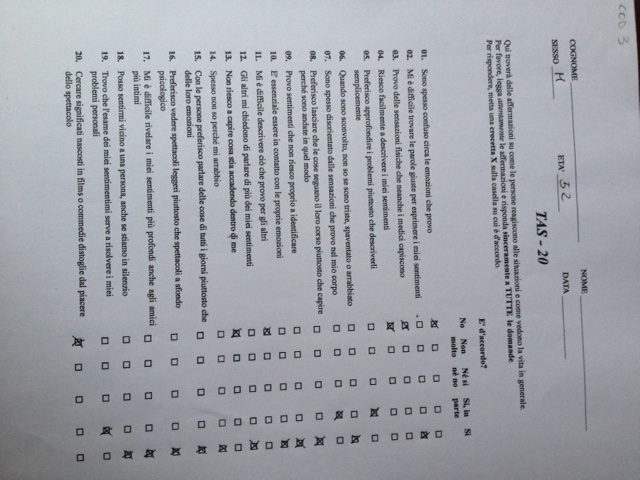

Supplement: Data S1 [file peerj-04-1864-s001.zip › gruppo clinico/IMG_3249.jpg]

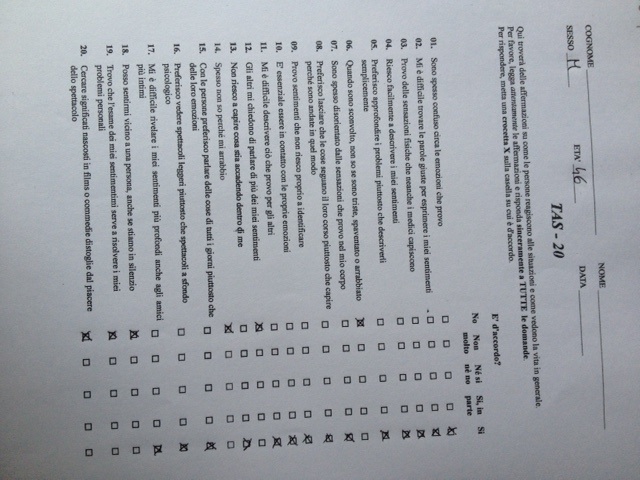

Supplement: Data S1 [file peerj-04-1864-s001.zip › gruppo clinico/IMG_3250.jpg]

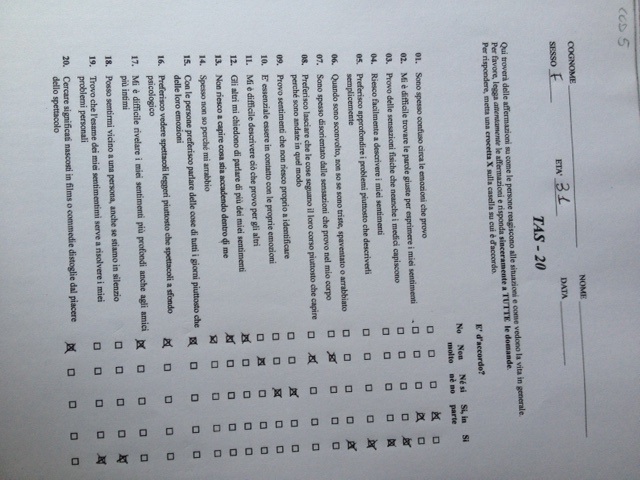

Supplement: Data S1 [file peerj-04-1864-s001.zip › gruppo clinico/IMG_3251.jpg]

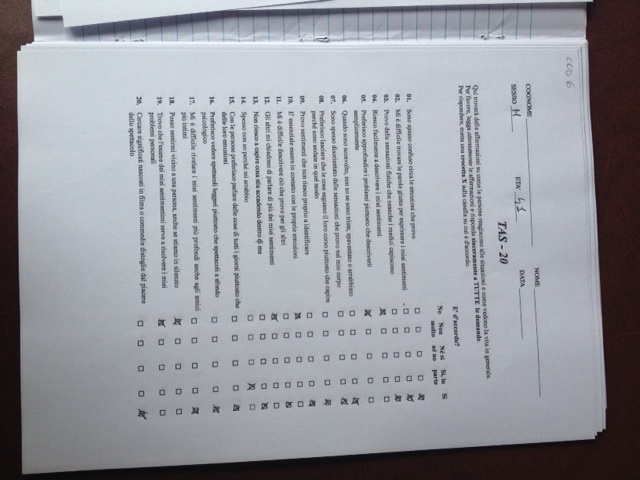

Supplement: Data S1 [file peerj-04-1864-s001.zip › gruppo clinico/IMG_3253.jpg]

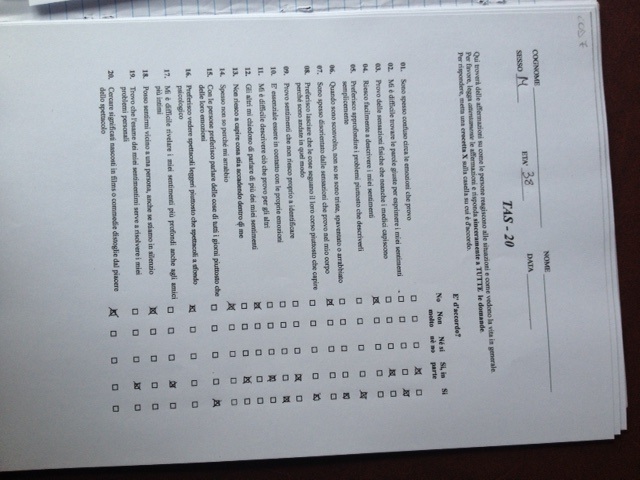

Supplement: Data S1 [file peerj-04-1864-s001.zip › gruppo clinico/IMG_3254.jpg]

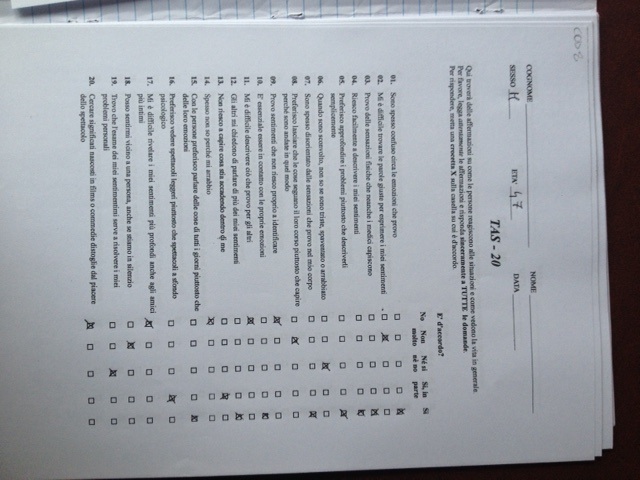

Supplement: Data S1 [file peerj-04-1864-s001.zip › gruppo clinico/IMG_3255.jpg]

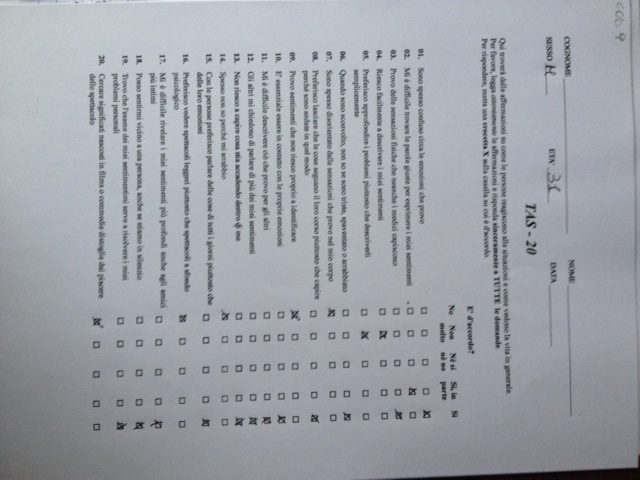

Supplement: Data S1 [file peerj-04-1864-s001.zip › gruppo clinico/IMG_3256.jpg]

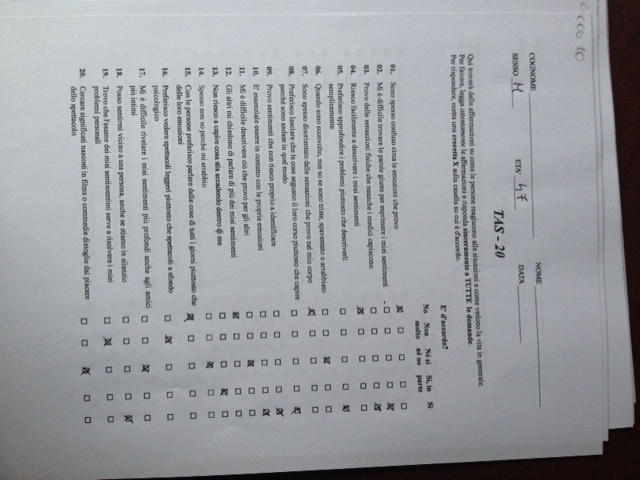

Supplement: Data S1 [file peerj-04-1864-s001.zip › gruppo clinico/IMG_3257.jpg]

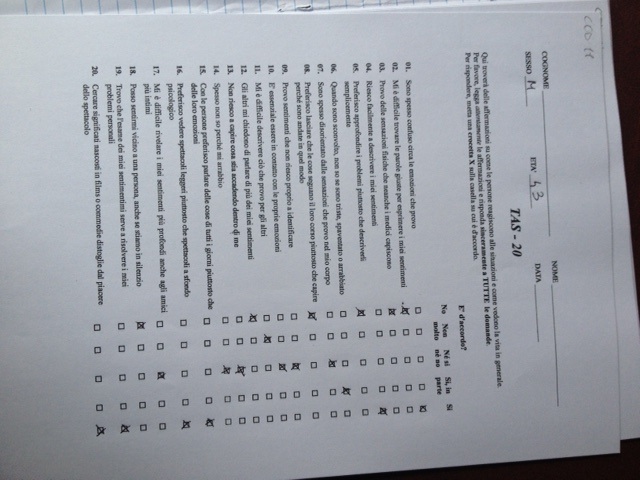

Supplement: Data S1 [file peerj-04-1864-s001.zip › gruppo clinico/IMG_3258.jpg]

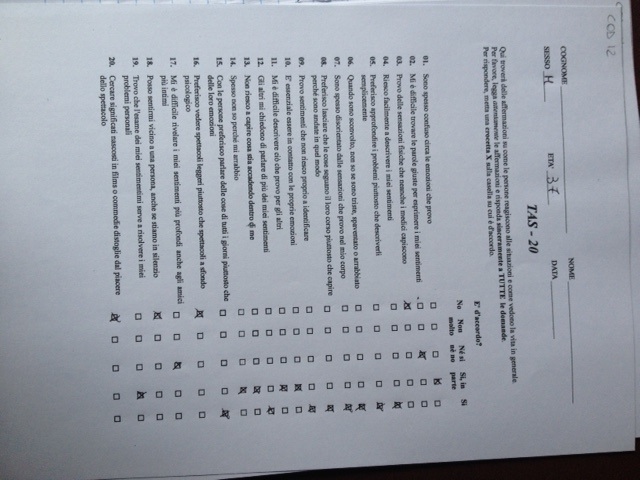

Supplement: Data S1 [file peerj-04-1864-s001.zip › gruppo clinico/IMG_3259.jpg]

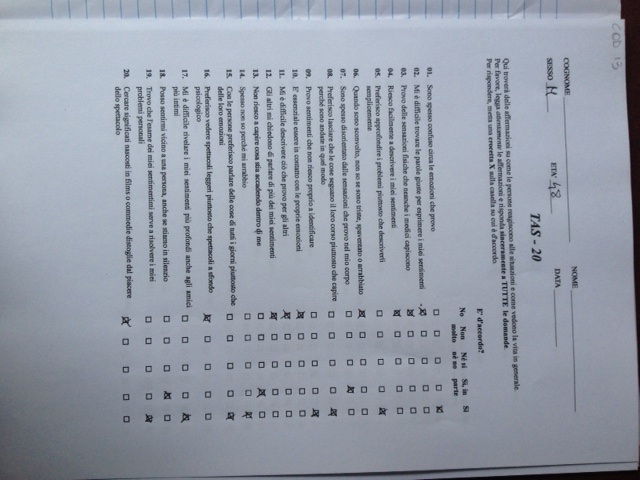

Supplement: Data S1 [file peerj-04-1864-s001.zip › gruppo clinico/IMG_3260.jpg]

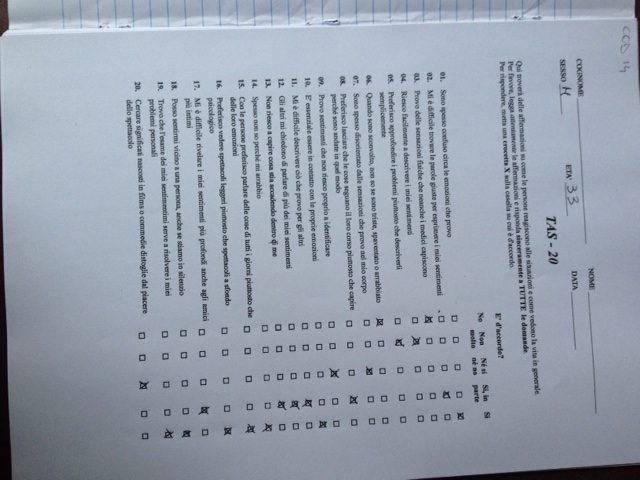

Supplement: Data S1 [file peerj-04-1864-s001.zip › gruppo clinico/IMG_3261.jpg]

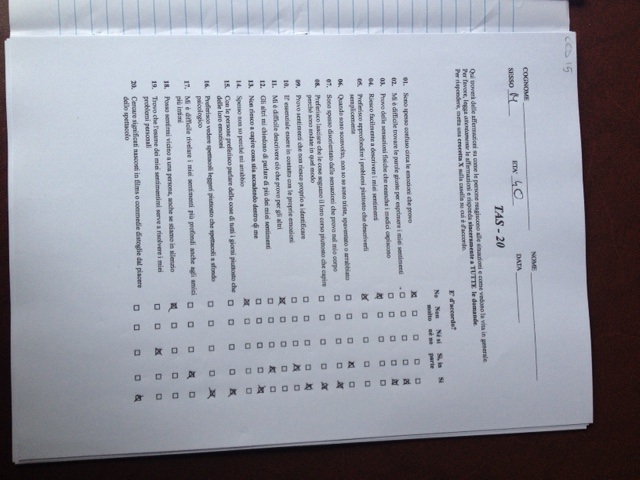

Supplement: Data S1 [file peerj-04-1864-s001.zip › gruppo clinico/IMG_3262.jpg]

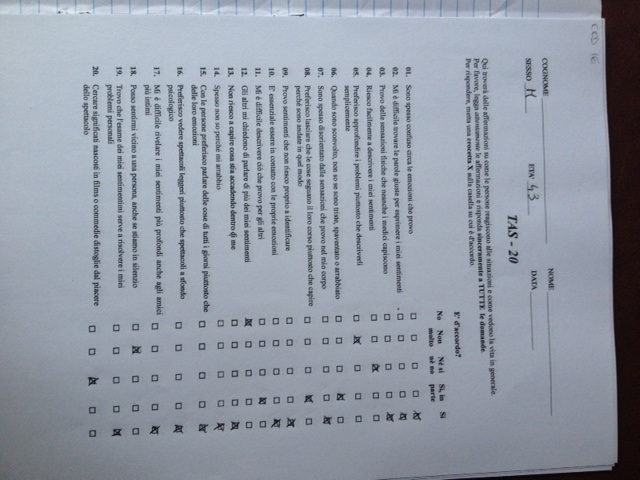

Supplement: Data S1 [file peerj-04-1864-s001.zip › gruppo clinico/IMG_3263.jpg]

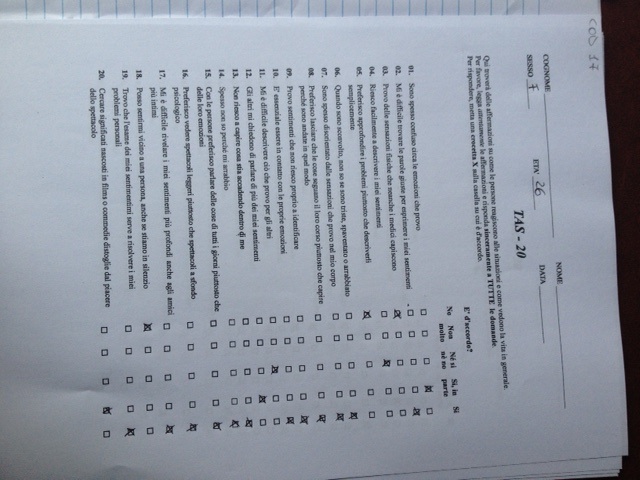

Supplement: Data S1 [file peerj-04-1864-s001.zip › gruppo clinico/IMG_3264.jpg]

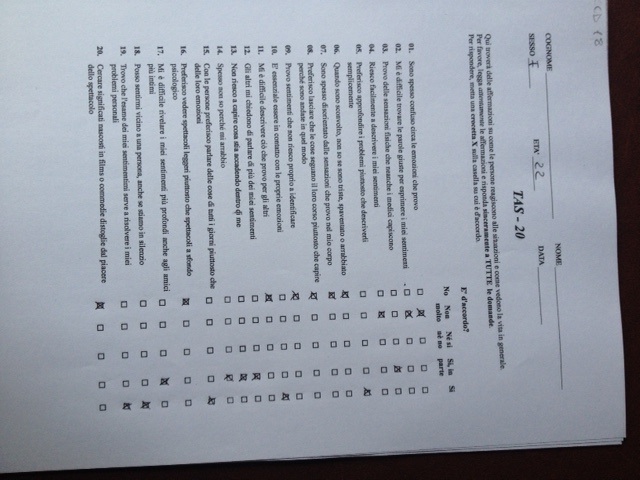

Supplement: Data S1 [file peerj-04-1864-s001.zip › gruppo clinico/IMG_3265.jpg]

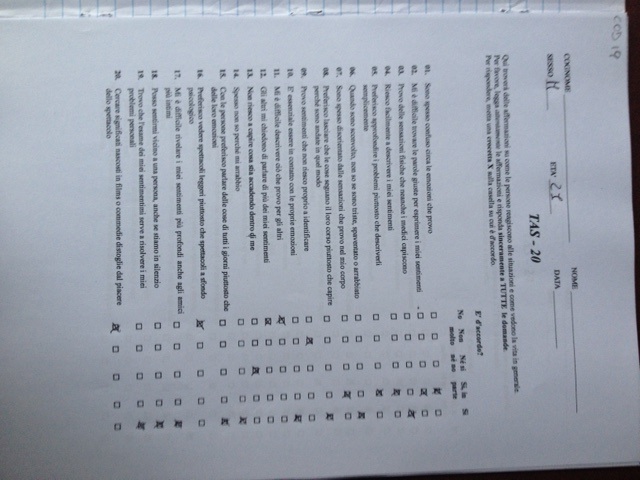

Supplement: Data S1 [file peerj-04-1864-s001.zip › gruppo clinico/IMG_3266.jpg]

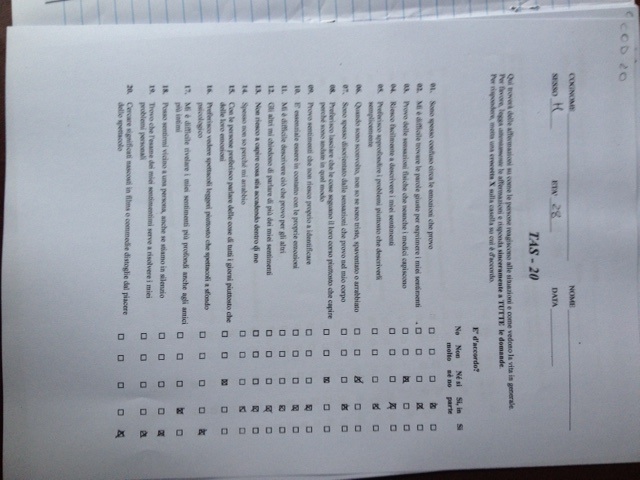

Supplement: Data S1 [file peerj-04-1864-s001.zip › gruppo clinico/IMG_3267.jpg]

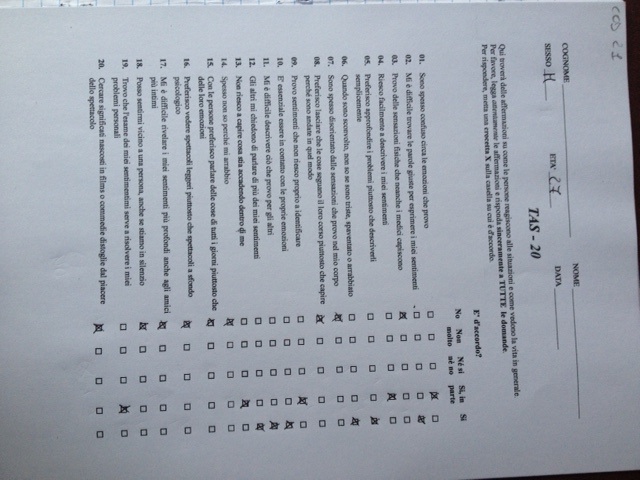

Supplement: Data S1 [file peerj-04-1864-s001.zip › gruppo clinico/IMG_3268.jpg]

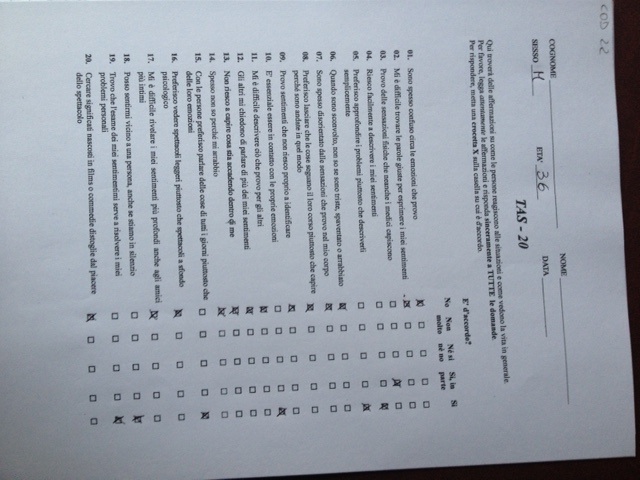

Supplement: Data S1 [file peerj-04-1864-s001.zip › gruppo clinico/IMG_3269.jpg]

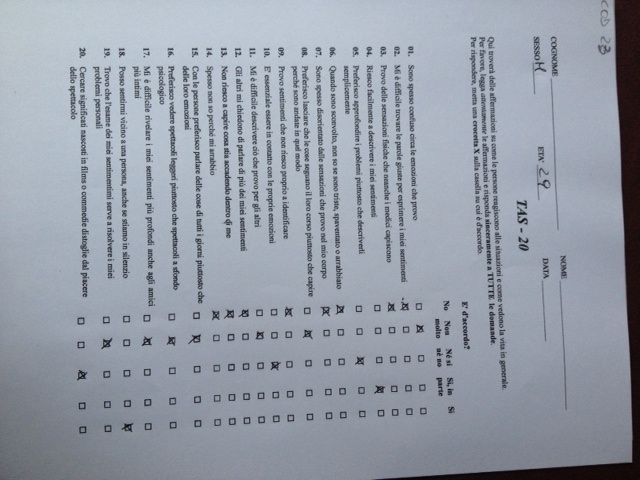

Supplement: Data S1 [file peerj-04-1864-s001.zip › gruppo clinico/IMG_3270.jpg]

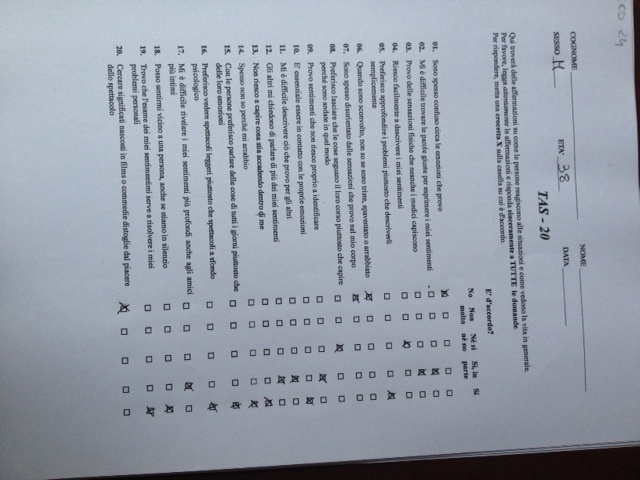

Supplement: Data S1 [file peerj-04-1864-s001.zip › gruppo clinico/IMG_3271.jpg]

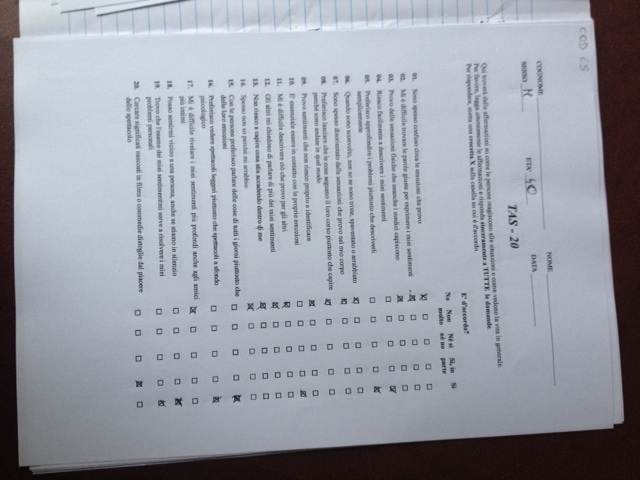

Supplement: Data S1 [file peerj-04-1864-s001.zip › gruppo clinico/IMG_3272.jpg]

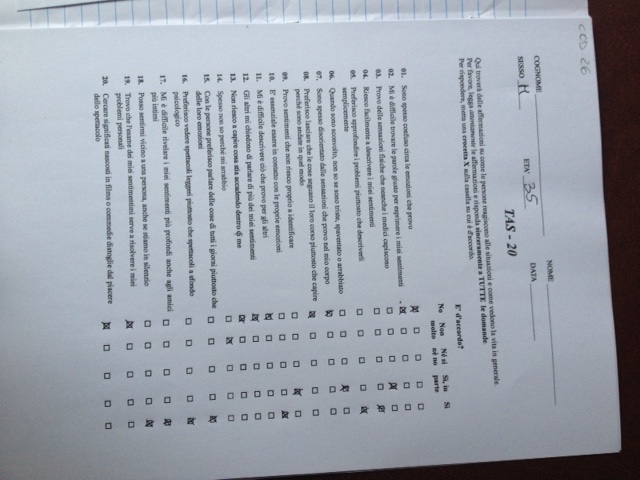

Supplement: Data S1 [file peerj-04-1864-s001.zip › gruppo clinico/IMG_3273.jpg]

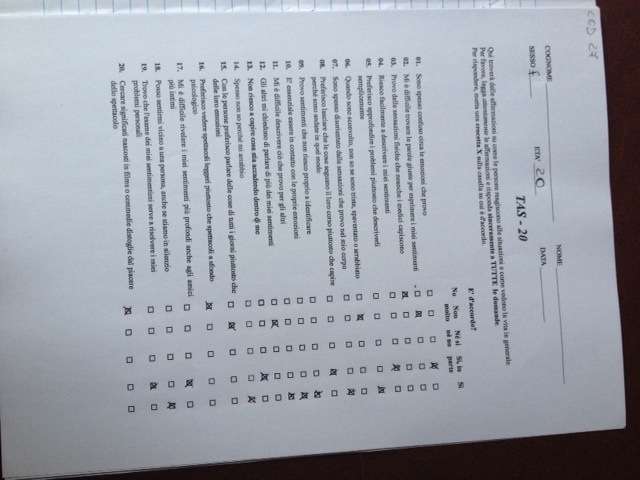

Supplement: Data S1 [file peerj-04-1864-s001.zip › gruppo clinico/IMG_3274.jpg]

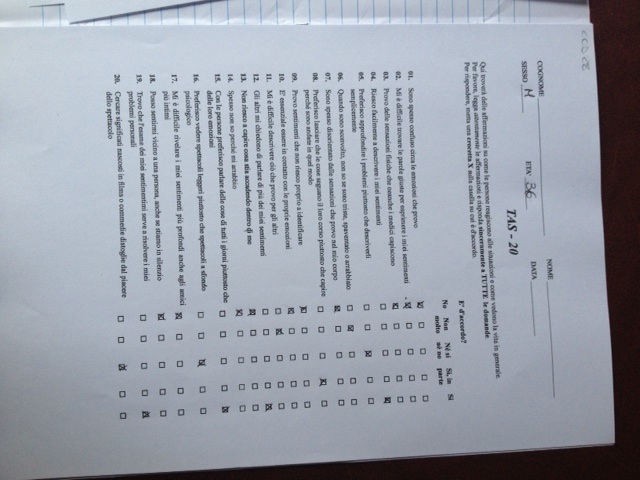

Supplement: Data S1 [file peerj-04-1864-s001.zip › gruppo clinico/IMG_3275.jpg]

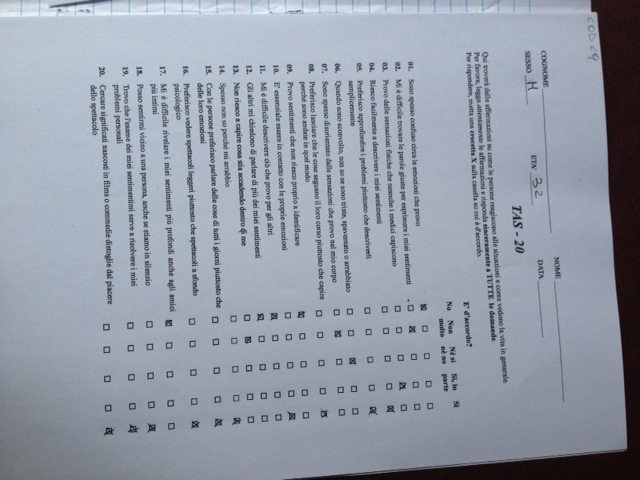

Supplement: Data S1 [file peerj-04-1864-s001.zip › gruppo clinico/IMG_3276.jpg]

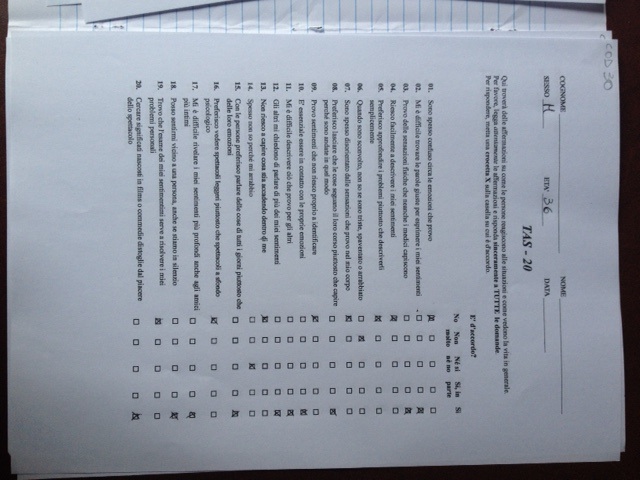

Supplement: Data S1 [file peerj-04-1864-s001.zip › gruppo clinico/IMG_3277.jpg]

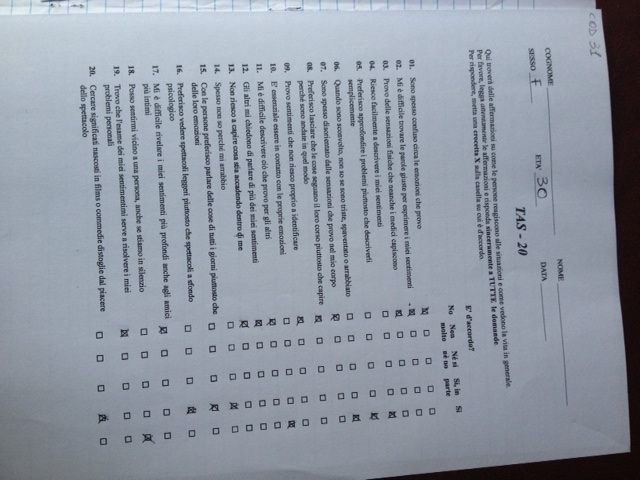

Supplement: Data S1 [file peerj-04-1864-s001.zip › gruppo clinico/IMG_3278.jpg]

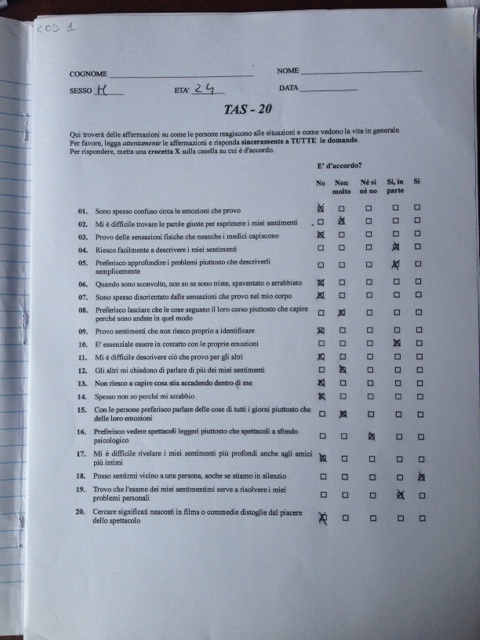

Supplement: Data S2 [file peerj-04-1864-s002.zip › gruppo controllo /IMG_3215.jpg]

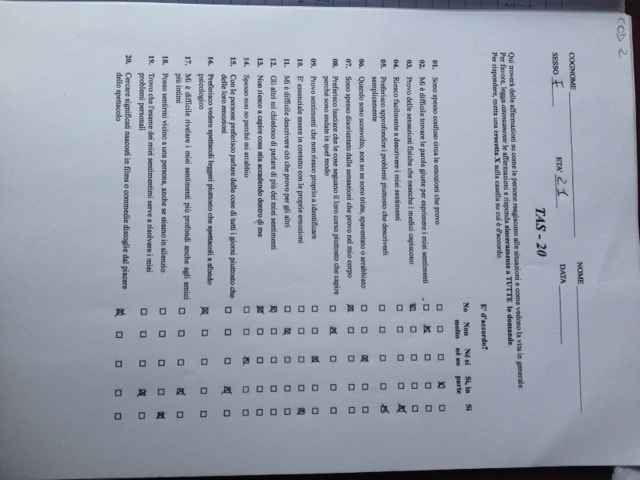

Supplement: Data S2 [file peerj-04-1864-s002.zip › gruppo controllo /IMG_3216.jpg]

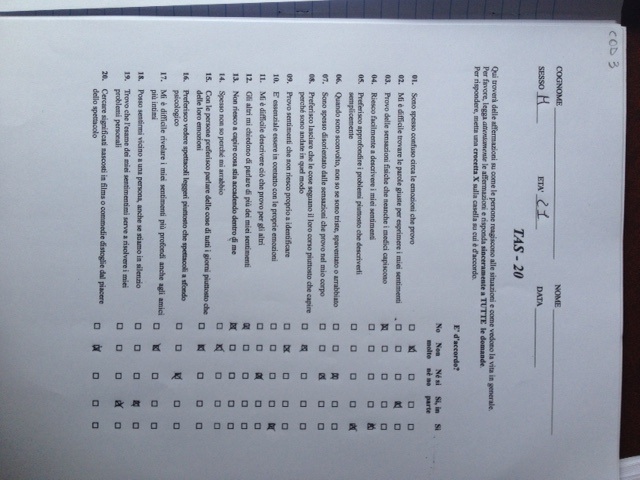

Supplement: Data S2 [file peerj-04-1864-s002.zip › gruppo controllo /IMG_3217.jpg]

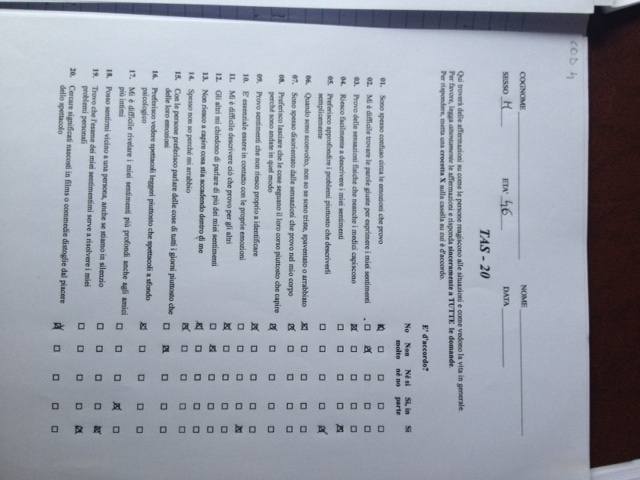

Supplement: Data S2 [file peerj-04-1864-s002.zip › gruppo controllo /IMG_3218.jpg]

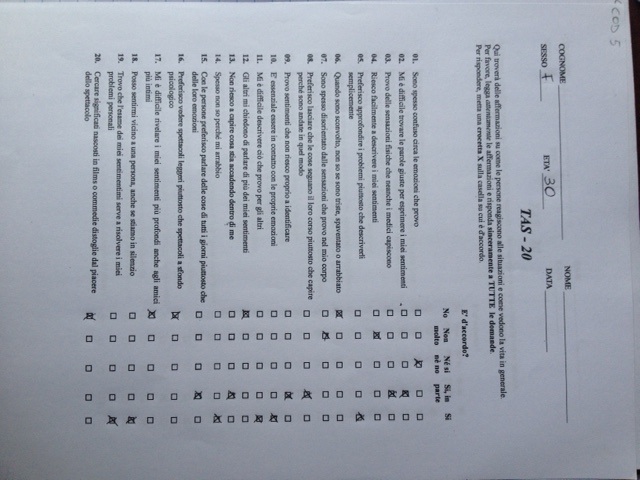

Supplement: Data S2 [file peerj-04-1864-s002.zip › gruppo controllo /IMG_3219.jpg]

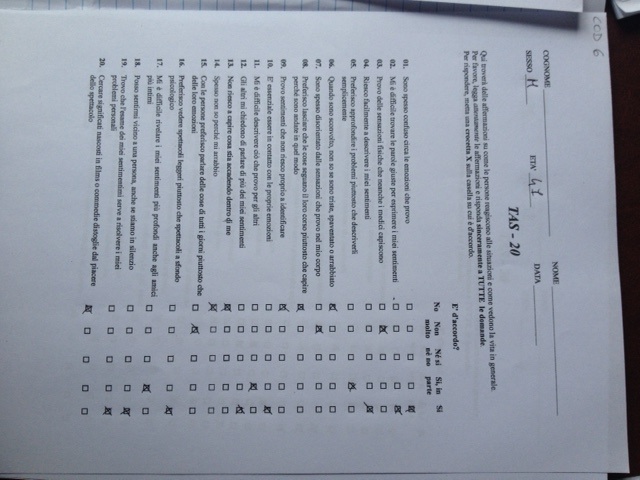

Supplement: Data S2 [file peerj-04-1864-s002.zip › gruppo controllo /IMG_3220.jpg]

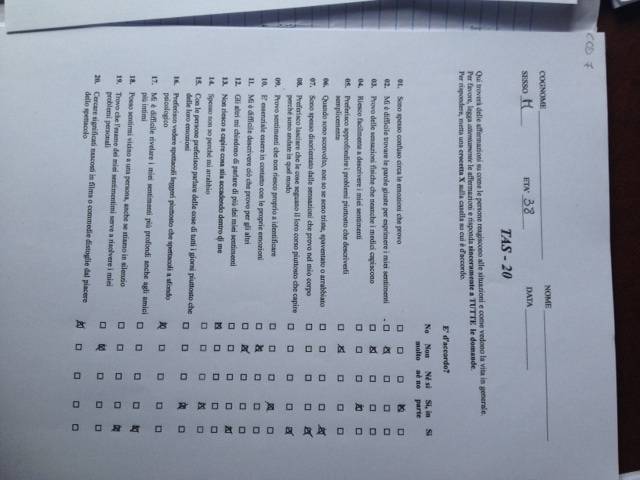

Supplement: Data S2 [file peerj-04-1864-s002.zip › gruppo controllo /IMG_3221.jpg]

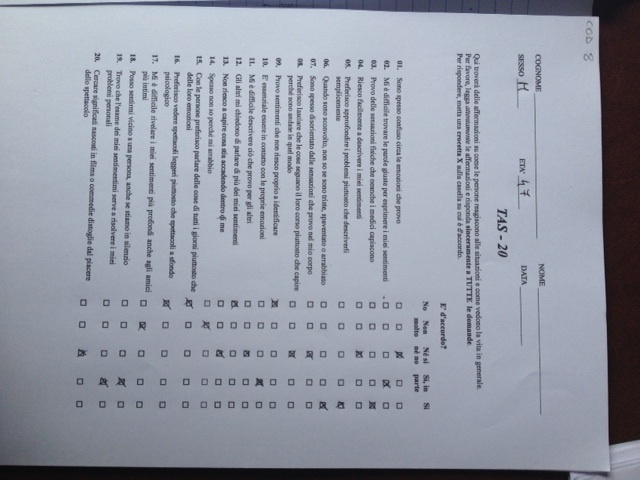

Supplement: Data S2 [file peerj-04-1864-s002.zip › gruppo controllo /IMG_3222.jpg]

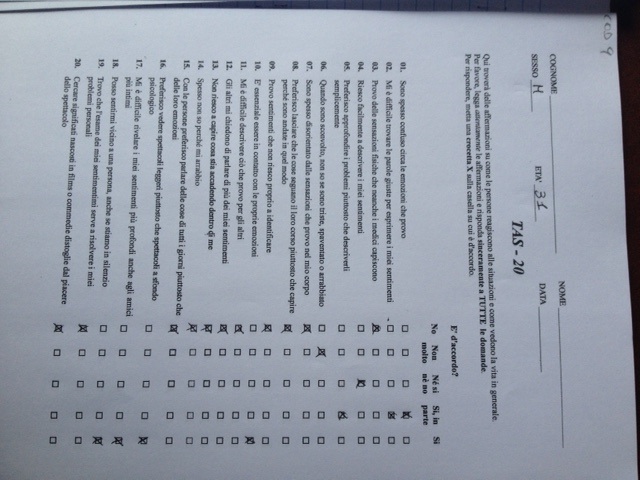

Supplement: Data S2 [file peerj-04-1864-s002.zip › gruppo controllo /IMG_3223.jpg]

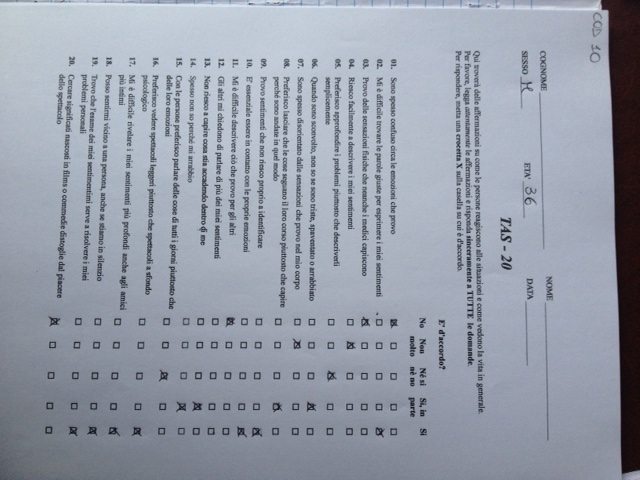

Supplement: Data S2 [file peerj-04-1864-s002.zip › gruppo controllo /IMG_3224.jpg]

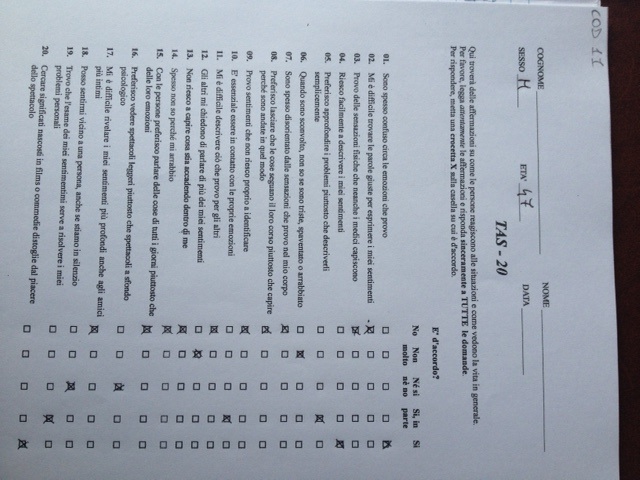

Supplement: Data S2 [file peerj-04-1864-s002.zip › gruppo controllo /IMG_3225.jpg]

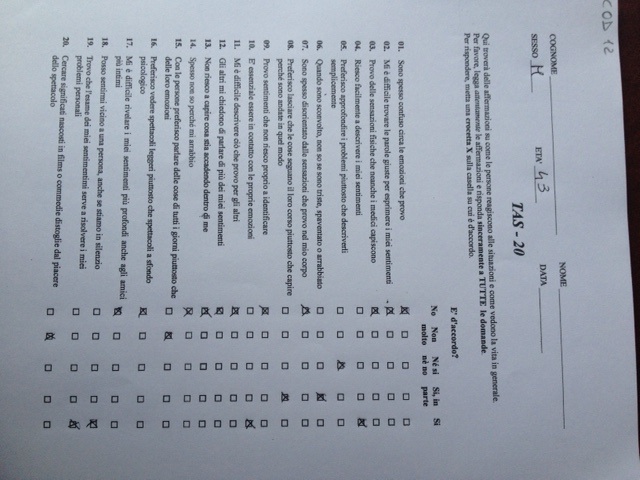

Supplement: Data S2 [file peerj-04-1864-s002.zip › gruppo controllo /IMG_3226.jpg]

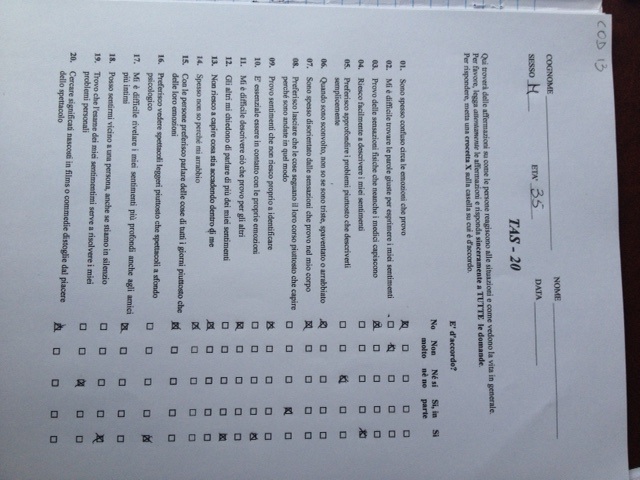

Supplement: Data S2 [file peerj-04-1864-s002.zip › gruppo controllo /IMG_3227.jpg]

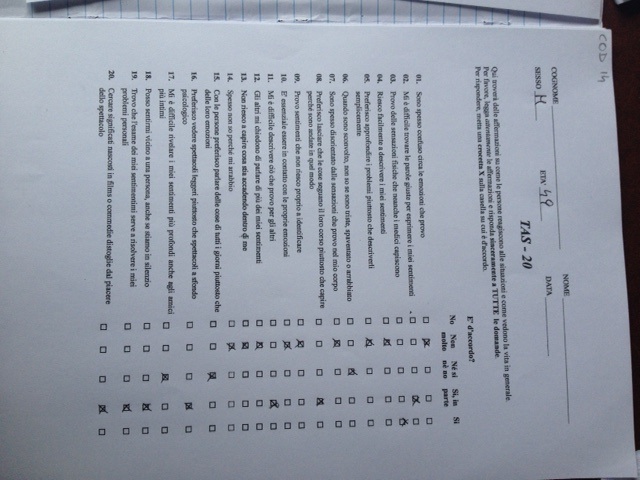

Supplement: Data S2 [file peerj-04-1864-s002.zip › gruppo controllo /IMG_3228.jpg]

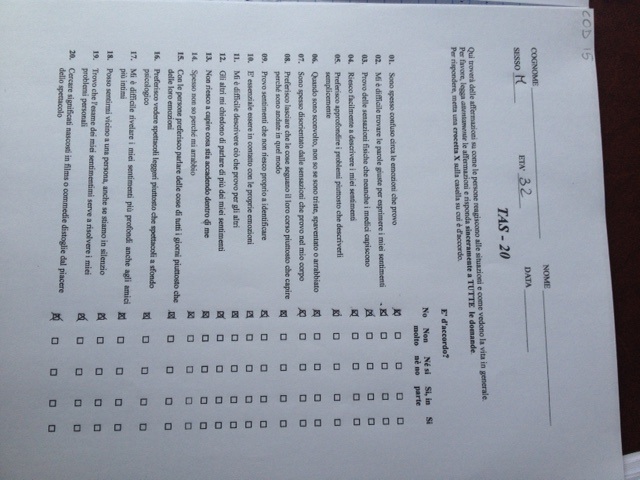

Supplement: Data S2 [file peerj-04-1864-s002.zip › gruppo controllo /IMG_3229.jpg]

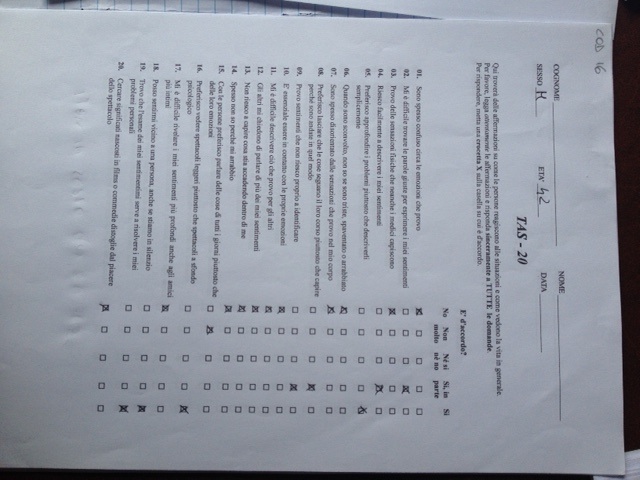

Supplement: Data S2 [file peerj-04-1864-s002.zip › gruppo controllo /IMG_3230.jpg]

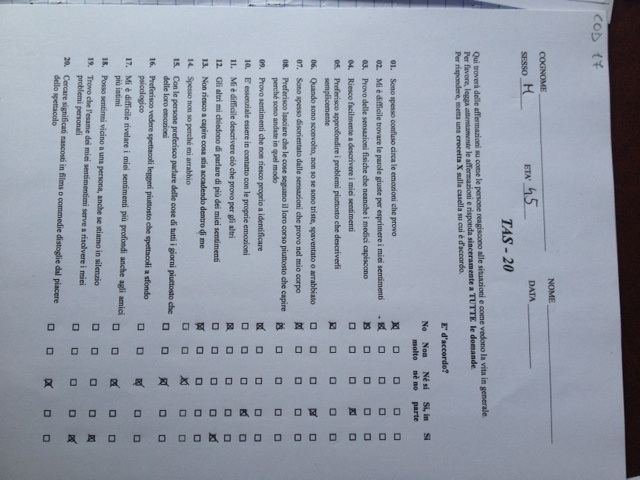

Supplement: Data S2 [file peerj-04-1864-s002.zip › gruppo controllo /IMG_3231.jpg]

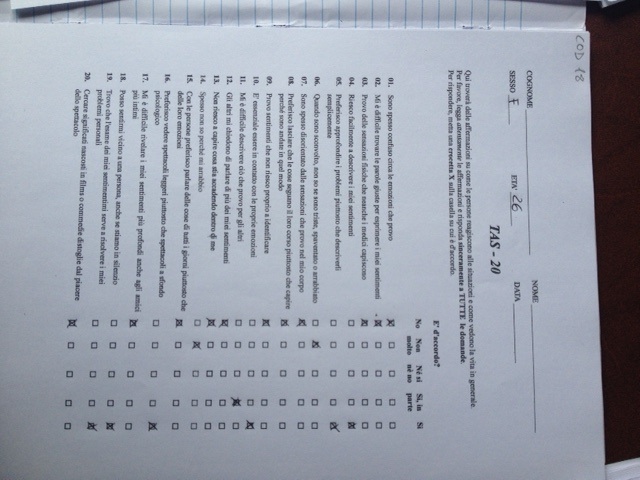

Supplement: Data S2 [file peerj-04-1864-s002.zip › gruppo controllo /IMG_3232.jpg]

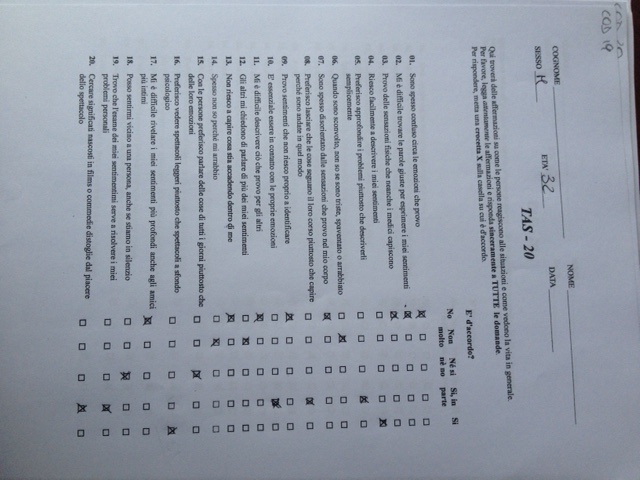

Supplement: Data S2 [file peerj-04-1864-s002.zip › gruppo controllo /IMG_3233.jpg]

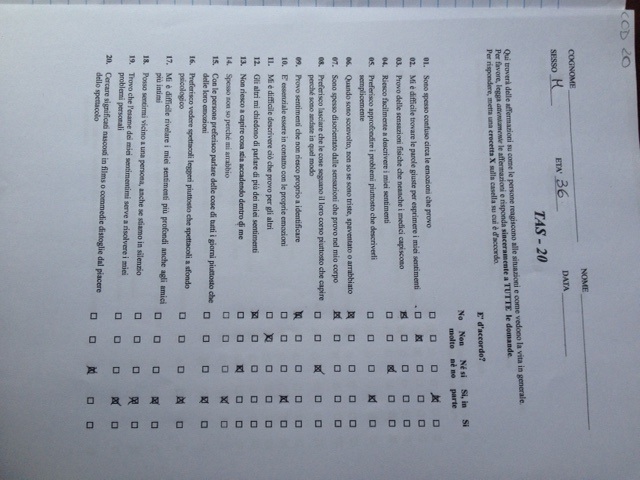

Supplement: Data S2 [file peerj-04-1864-s002.zip › gruppo controllo /IMG_3234.jpg]

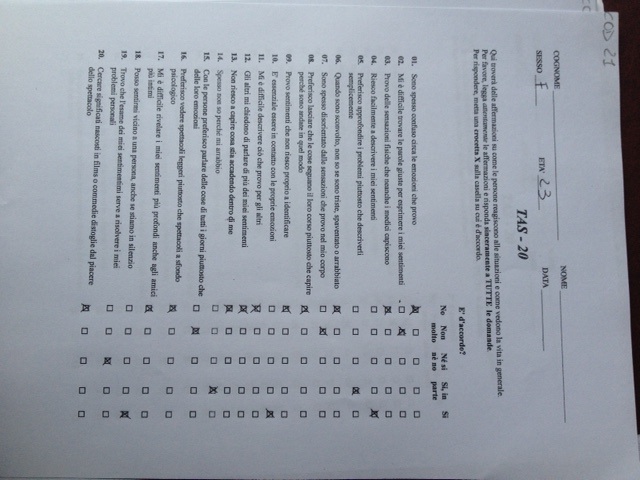

Supplement: Data S2 [file peerj-04-1864-s002.zip › gruppo controllo /IMG_3235.jpg]

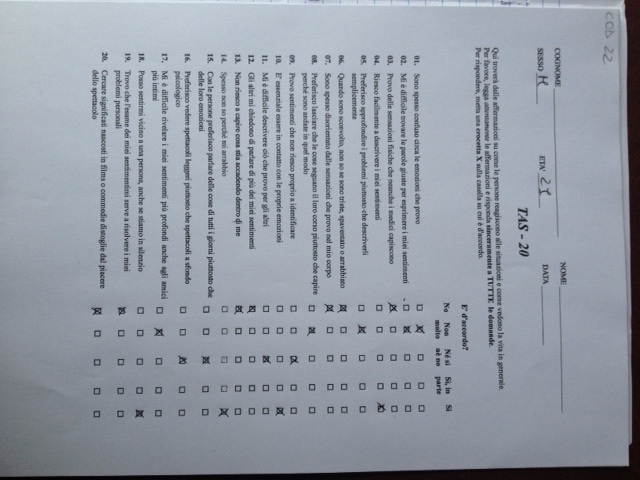

Supplement: Data S2 [file peerj-04-1864-s002.zip › gruppo controllo /IMG_3236.jpg]

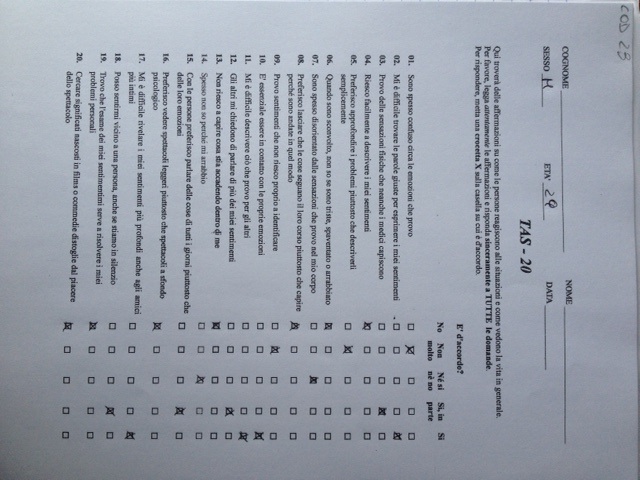

Supplement: Data S2 [file peerj-04-1864-s002.zip › gruppo controllo /IMG_3237.jpg]

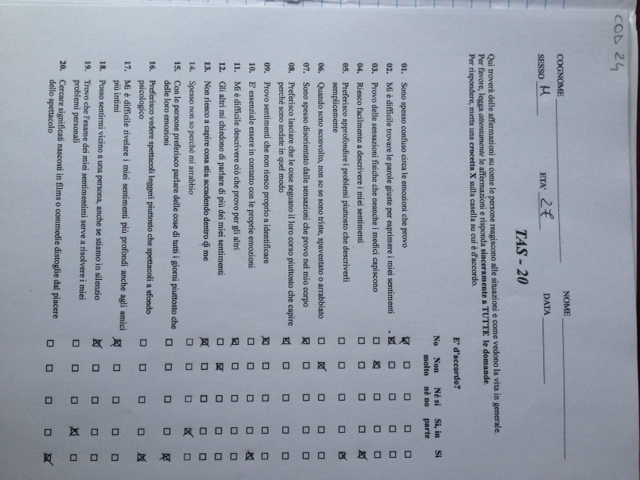

Supplement: Data S2 [file peerj-04-1864-s002.zip › gruppo controllo /IMG_3238.jpg]

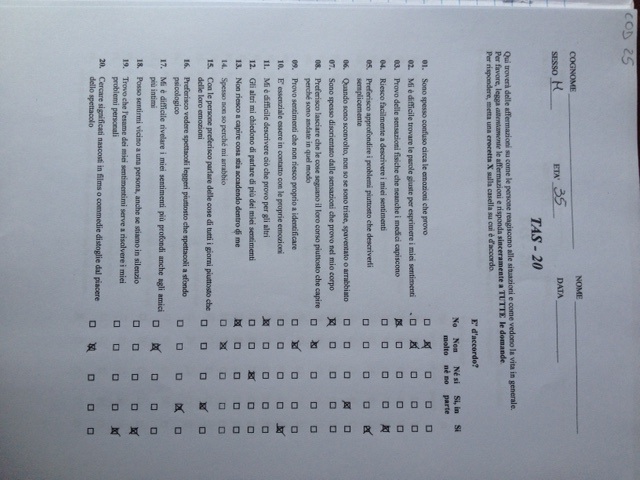

Supplement: Data S2 [file peerj-04-1864-s002.zip › gruppo controllo /IMG_3239.jpg]

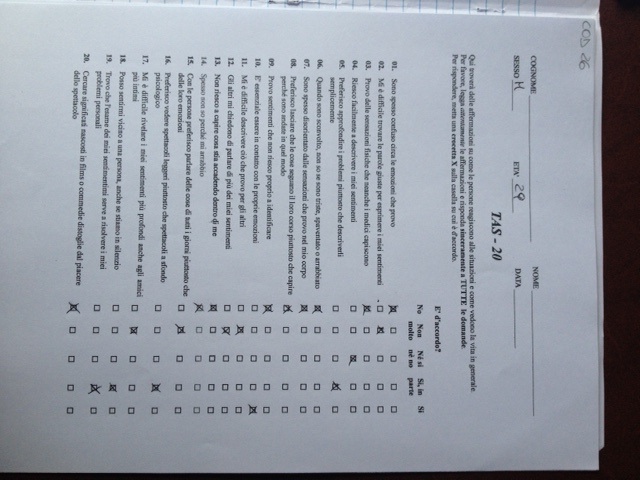

Supplement: Data S2 [file peerj-04-1864-s002.zip › gruppo controllo /IMG_3240.jpg]

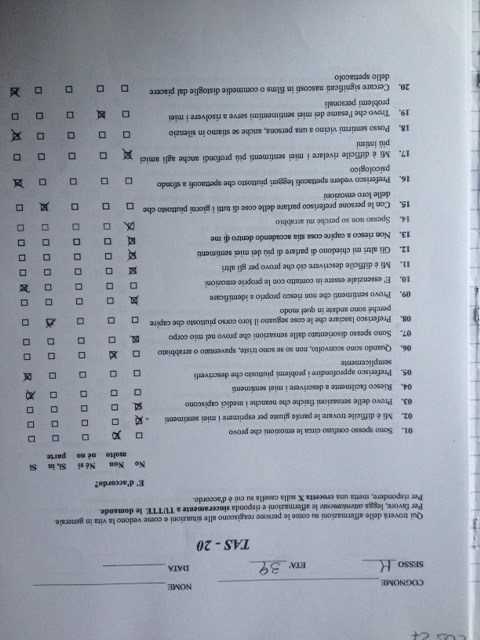

Supplement: Data S2 [file peerj-04-1864-s002.zip › gruppo controllo /IMG_3241.jpg]

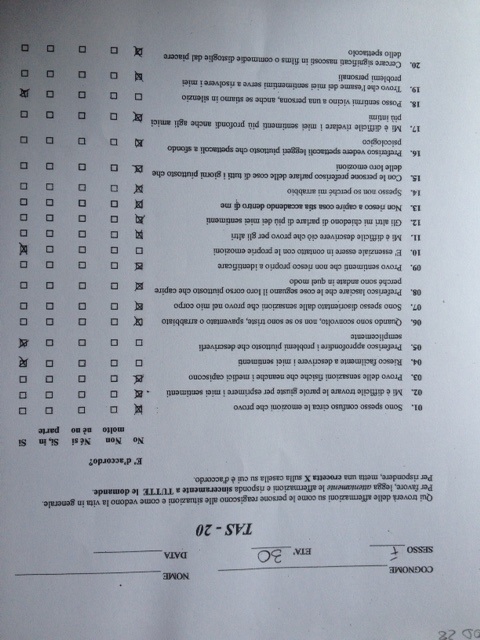

Supplement: Data S2 [file peerj-04-1864-s002.zip › gruppo controllo /IMG_3242.jpg]

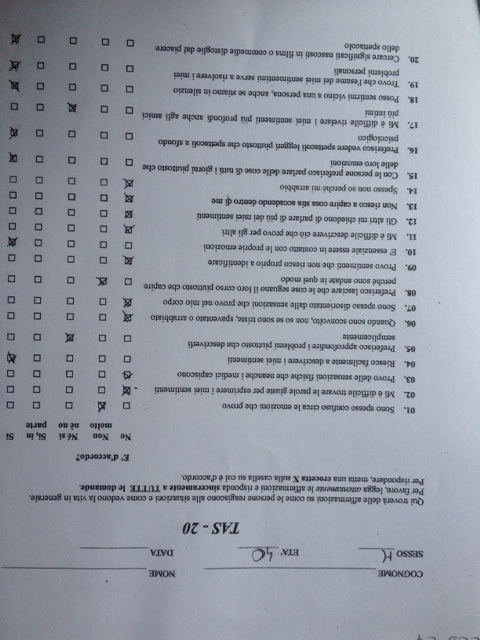

Supplement: Data S2 [file peerj-04-1864-s002.zip › gruppo controllo /IMG_3243.jpg]

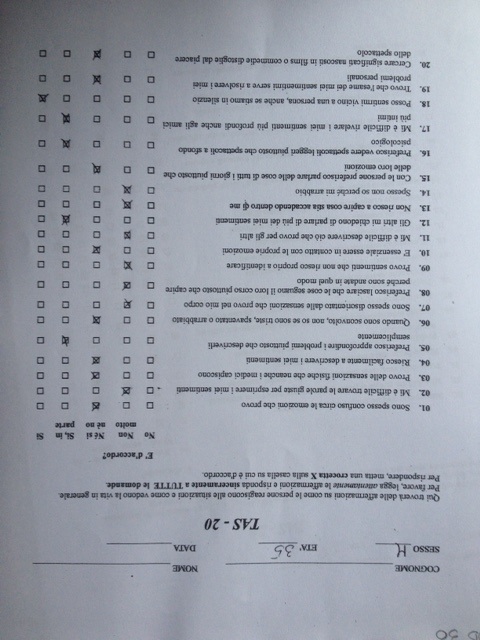

Supplement: Data S2 [file peerj-04-1864-s002.zip › gruppo controllo /IMG_3244.jpg]

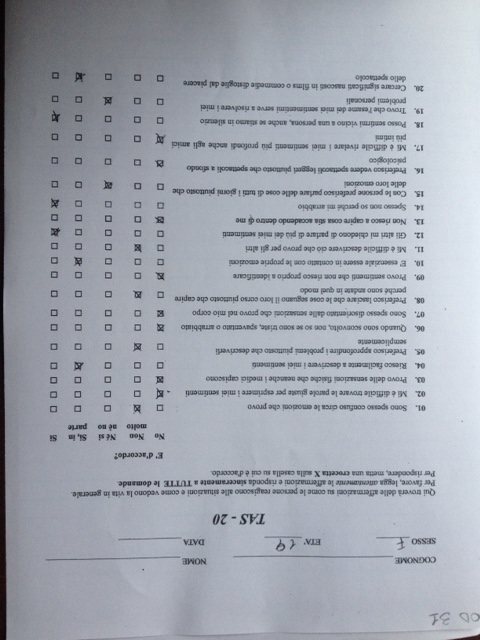

Supplement: Data S2 [file peerj-04-1864-s002.zip › gruppo controllo /IMG_3245.jpg]
